# A Mathematical Model of Skeletal Muscle Disease and Immune Response in the *mdx* Mouse

**DOI:** 10.1155/2014/871810

**Published:** 2014-06-11

**Authors:** Abdul Salam Jarrah, Filippo Castiglione, Nicholas P. Evans, Robert W. Grange, Reinhard Laubenbacher

**Affiliations:** ^1^Department of Mathematics and Statistics, American University of Sharjah, Sharjah 26666, UAE; ^2^Institute for Applied Mathematics, National Research Council of Italy, 00185 Rome, Italy; ^3^Department of Population Health Sciences, Virginia Tech, Blacksburg, VA 24060, USA; ^4^Department of Human Nutrition, Foods and Exercise, Virginia Tech, Blacksburg, VA 24060, USA; ^5^Center of Quantitative Medicine, University of Connecticut Health Center, Farmington, CT 06030, USA; ^6^Jackson Laboratory for Genomic Medicine, Farmington, CT 06030, USA

## Abstract

Duchenne muscular dystrophy (DMD) is a genetic disease that results in the death of affected boys by early adulthood. The genetic defect responsible for DMD has been known for over 25 years, yet at present there is neither cure nor effective treatment for DMD. During early disease onset, the * mdx mouse *has been validated as an animal model for DMD and use of this model has led to valuable but incomplete insights into the disease process. For example, immune cells are thought to be responsible for a significant portion of muscle cell death in the * mdx *mouse; however, the role and time course of the immune response in the dystrophic process have not been well described. In this paper we constructed a simple mathematical model to investigate the role of the immune response in muscle degeneration and subsequent regeneration in the * mdx *mouse model of Duchenne muscular dystrophy. Our model suggests that the immune response contributes substantially to the muscle degeneration and regeneration processes. Furthermore, the analysis of the model predicts that the immune system response oscillates throughout the life of the mice, and the damaged fibers are never completely cleared.

## 1. Background

Duchenne muscular dystrophy (DMD) is a lethal, X-chromosome muscle wasting disease affecting approximately one in 3,500 boys [1, 2]. Patients appear clinically normal at birth with the exception of elevated serum creatine kinase levels. The onset of DMD begins in early childhood with the first observed symptoms between two and five years of age. Typically by the age of 12, DMD patients require the use of a wheelchair due to the loss of lower limb muscle strength. Progressive weakness of the arms and legs, along with kyphoscoliosis, continues through late disease progression. Many patients die in their late teens or early twenties due to respiratory or cardiac complications [1, 3]. Currently, there are no effective means of therapy or treatment for DMD.

In 1984, Bulfield et al. identified a spontaneous mutation in C57BL/10ScSn inbred mice that exhibited a disease state similar to human DMD [4]. The X chromosome-linked mutation resulted in mice (*mdx* mice) with high serum levels of muscle enzymes and with histological lesions comparable to those seen in human muscular dystrophy. This mutation in the murine dystrophin gene caused an absence of dystrophin in skeletal muscle and this key defect validated the* mdx* mouse as a suitable model of the early onset of DMD human disease [5, 6].

The histology and time course of the disease in* mdx* mouse model are very different from those in DMD patients: relatively normal life span and overall fitness compared to progressive physical impairment leading to death in DMD patients [7]. Nonetheless, the* mdx* mouse model is regarded as the best animal model, especially of the early onset of DMD.

Dystrophin deficiency does not always produce muscle degeneration at all life stages, in all muscle phenotypes, or in all animal models [8]. In dystrophin-deficient skeletal muscle, for example, mechanical injury and proteolysis may be important factors but do not fully explain DMD pathogenesis. Mechanisms such as the immune/inflammatory response to injury appear to contribute substantially to muscle pathophysiology. Observations of activated immune cell infiltrates in dystrophic muscle suggest that the immune/inflammatory response may play a role in exacerbating the disease [8–12].

Immune/inflammatory-mediated mechanisms, which result in muscle cell death and/or mechanisms leading to fibrosis, may be important initiators of lesions in dystrophin-deficient muscle. Large populations of lymphocytes, macrophages, and neutrophils are present in DMD muscle tissue [10]. T-cells and macrophages are classically thought to be responsible for triggering and orchestrating the immune response, inducing target cell death, recognizing immune stimuli, and removing cellular debris. Immunosuppressive therapy, such as treatment with glucocorticoids, improves muscle strength and prolongs ambulation in DMD patients but does not prevent disease progression [3, 12]. The article [13] is a comprehensive literature review of the immune-mediated molecular and signaling mechanisms that regulate the time course of the disease and the* mdx* mouse model.

One limitation found in the DMD literature is that there are few time course datasets that are consistent for muscle type, ages, or collected with the same methods. To our knowledge, there is not a time course dataset that accounts for the aspects of the immune response, muscle degeneration, and muscle regeneration as modeled herein.

In this paper we presented a simple mathematical model to investigate the role of the immune response in muscle degeneration in* mdx* mice. The mathematical model represents a novel approach to study DMD pathogenesis and to identify potential therapeutic targets. Using the available data, we constructed a mechanistic differential equations model as the first step toward building a comprehensive model of the immune response in DMD aiming to provide insight into the nature of the immune/inflammatory mechanisms contributing to DMD pathogenesis in the early disease stages. Understanding these underlying mechanisms will provide a key tool to develop effective therapeutic approaches.

The model incorporates the generally held hypothesis that the immune response contributes to muscle tissue damage via CD8+ T-cells that are recruited by macrophages through CD4+ T-cells. This simple model fits the available experimental time course data found in the literature. Moreover, the model suggests that CD8+ T-cells likely contribute to muscle damage and predicts two distinct modes for the long-term dynamics of the immune response.

## 2. Experimental Techniques and Data

Immune and histopathological time course data used for the model were obtained from available literature. We combined time course data for several different dystrophic muscles for the age range 14–84 days, including those for the concentrations of CD8+ and CD4+ T-cells in quadriceps [9], macrophages in soleus [11], procion orange dye uptake (as an indicator of fiber damage) in* soleus (SOL)* and histopathology in soleus and extensor* digitorum longus* (EDL) muscles [15], and TA muscles [14]. It should be noted here that the* extensor digitorum longus* (EDL) and* tibialis anterior* (TA) muscles are primarily fast twitch muscles whereas the* soleus* (SOL) is a slow twitch muscle.

Throughout this paper, we define* normal* muscle fibers as those that do not exhibit damage; that is, they are neither degenerating nor necrotic, nor do they demonstrate uptake of procion orange dye.* Damaged* fibers are those that are degenerating or necrotic.* Regenerating* fibers are those that were damaged and undergoing repair (identified by the presence of centralized nuclei).

## 3. The Mathematical Model

The model presented here describes in mathematical terms the action of the immune system on muscle tissues subsequent to damage triggered by a not well-specified mechanism. Since we are interested in following the role of the immune system during disease progression, we use a simplistic description of the development of damaged tissue while we model the cell interaction during the buildup of the adaptive (i.e., specific) immune response in more detail. In particular, we include macrophages that trigger T-helper activation enabling cytotoxicity for muscle cells. Whereas macrophages are employed in the removal of damaged cells, favoring tissue regeneration, they also stimulate specific CD8+ lymphocytes that in turn create additional damage. The model is based on the following set of variables: the concentration of immune cells (CD4+, CD8+ T-cells, and macrophages) and the fractions of morphologically normal muscle fibers, damaged muscle fibers, and regenerating muscle fibers in the muscle tissue.

The model variables are immune cells (cell numbers in a cubic millimeter of muscle tissue), macrophages (*M*) CD4+ T-helper lymphocytes (*H*) and CD8+ cytotoxic T lymphocytes (*C*), and* muscle fibers* (percentage of the whole muscle tissue): normal (*N*), damaged (*D*), and regenerating fibers (*R*).

The model equations are as follows:

(1) $$\begin{array}{c} \frac{dH}{dt}=b_{H}+k_{1}DM-d_{H}H, \end{array}$$

(2) $$\begin{array}{c} \frac{dC}{dt}=b_{C}+k_{2}DH-d_{C}C, \end{array}$$

(3) $$\begin{array}{c} \frac{dM}{dt}=b_{M}+k_{3}MD-d_{M}M, \end{array}$$

(4) $$\begin{array}{c} \frac{dN}{dt}=k_{4}R-k_{5}CN-\alpha N, \end{array}$$

(5) $$\begin{array}{c} \frac{dD}{dt}=k_{5}CN+\alpha N-k_{6}DM-d_{D}D, \end{array}$$

(6) $$\begin{array}{c} \frac{dR}{dt}=k_{6}DM+d_{D}D-k_{4}R. \end{array}$$

Here *α* = *α*(*t*) represents mechanical damage as a lognormal function of time. This damage triggers the cascade of events starting with macrophage infiltration into the tissue, recruitment of CD4+ cells, and activation of CD8+ cells. The choice of a lognormal function to model muscle cell failure comes from considering the accumulation of damage in the muscle tissue as a multiplicative degradation process. In brief, the degradation process leading to a lognormal model is obtained when the amount of degradation or damage that ends in complete failure follows the relationship *y*_*t*_ = (1 − *ε*_*t*_)*y*_*t*−1_, where *ε*_*t*_ are small independent random shocks (in our case occurring to each single muscle cell) during progression to failure. This relationship means that the increase in the amount of damage from one time point to the next is a small multiple of the total amount of damage accumulated (i.e., fewer undamaged muscle cells have to share the same muscle workload, resulting in a larger effort or stress that yields increased damage at the next time point). When a large number of cells are damaged, by the central limit theorem, the probability of failure can be approximated by a lognormal distribution, or, to be more precise, since failure occurs when the amount of degradation reaches a critical point, the time to failure will be modeled by a lognormal [16],

(7) $$\begin{array}{c} \alpha \left(t\right)=\frac{h}{t\sigma \sqrt{2\pi }}e^{-{\left(\ln\left(t\right)-m\right)}^{2}/\left(2\sigma^{2}\right)}, \end{array}$$

where *m*, *h*, and *σ* are parameters to be determined on the basis of the available experimental data.

For this model, we assume that the physiological damage arising from regular muscular activity is responsible for the initial mechanical damage. Due to genetics and other factors that are determinant in developing the disease, this initial damage is amplified in the* mdx* mice leading to a strong immune response, while in the wild-type mice (disease-free) the initial damage will not be amplified resulting in a balanced immune response. We model this assumption by simply assuming that, in the wild-type model, the amplitude *h*, which is proportionally related to the mechanical damage, is negligible with respect to its counterpart in* mdx* mice.

Equations (1)–(3) represent the rate of change of the immune cell counts of CD4+ (*H*) and CD8+ T cells (*C*) and macrophages (*M*), respectively. In particular, (2) shows that lymphocytes CD8+ T-cells are replenished at a constant rate *b*_*C*_ and die at the rate *d*_*C*_ *C*. The term *k*_2_ *DH* stands for the activation of CD8+ cells from damaged cells, but only in presence of helper T-cells which activate them. Similarly the term *k*_1_ *DM* in (1) describes the activation of CD4+ T-cells by macrophages in the presence of damage. Alternatively macrophages in (3) may also be activated by the damage alone (i.e., do not require a second signal).

Equations (4)–(6) represent the rate of change of the percentage of morphologically normal (*N*) damaged (*D*) and regenerating (*R*) muscle fibers. Equation (5) reflects the assumption that the damage accumulates according to the multiplicative degradation process described above with a time-to-failure *α* = *α*(*t*) modeled as a lognormal (term −*αN* in (4), and the respective term *αN* in (5)).

Damaged cells are either removed by the macrophages at rate *k*_6_ *DM* or by other mechanisms (unspecified) at the rate *d*_*D*_ *D*. Equation (4) indicates that normal cells are replenished from regenerating fibers at the rate *k*_4_ *R* and are damaged at the rate *k*_5_ *CN* by the presence of specific CD8+ T-cells.

Note that (6) can be rewritten as *dR*/*dt* = −(*dN*/*dt* + *dD*/*dt*), which is the assumption that a muscle fiber can be either normal, damaged, or regenerating while the total number of fibers in a muscle remains constant.

The model assumes that if there are no damaged muscle fibers in the tissue, the numbers of different immune cells (i.e., CD8+, CD4+ T-cells, and macrophages) do not change. Furthermore, the assumption that the muscle tissue initially has no damaged or regenerating fibers implies the following initial baselines: *N*(0) = 100 and *D*(0) = *R*(0) = 0.

### 3.1. Parameter Estimation

In addition to the cell counts at time zero for the three types of immune cells, there are 13 parameters in the model. As nothing is known about the values of these parameters in the literature, especially in a muscle tissue, we estimated their values by fitting, using the freely available software* COPASI* [17]. COPASI is a stand-alone software for the simulation and analysis of network models and their dynamics. It has many functions, including parameter estimation and sensitivity analysis, both of which we used throughout this study. The optimal parameter values presented in Table 1 were obtained by using particle swarm and the genetic algorithms to search for a global solution. Hooke-Jeeves and Lavenberg-Marquardt algorithms were used to find the best local solution. All of these and many other known optimization algorithms are already implemented in COPASI. We used the immune response experimental data from [9, 11] as well as the muscles data from [14, 15] to estimate the parameters of the model.

We assume that the cell count of any type of the immune cells at time zero in* mdx* mice is equal to the cell count of that type of immune cell in a wild-type mouse. Based on the available data in the literature about wild-type C57 mice [15], we assumed that there are initially no CD4+ T-cells, and we set the initial number of macrophages in the tissue to 400 cells and the number of CD8+ T-cells to 4. It should be noted here that the model is not sensitive, however, to any of the initial counts of the immune cells, as we will explain in the next section.

### 3.2. Model Assessment

**Figure 1:**
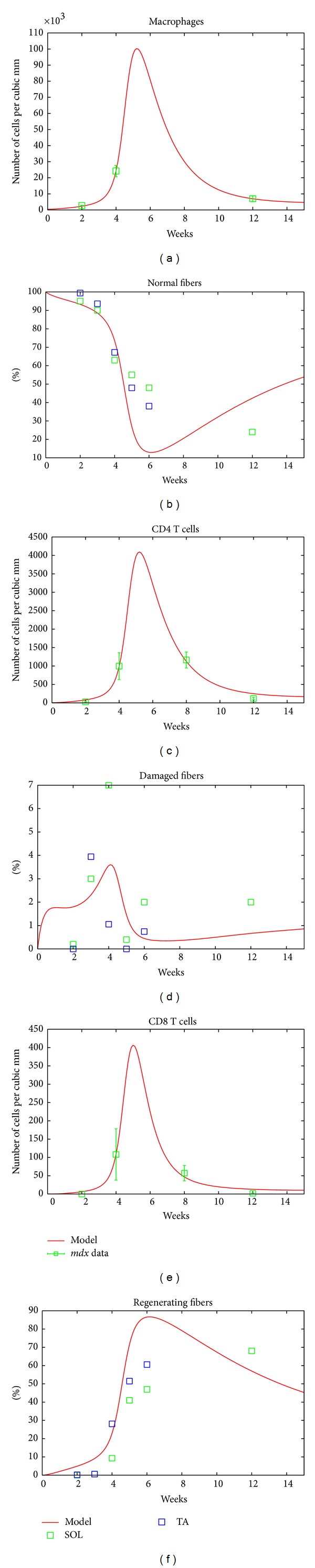
* mdx* mice. The left panel shows the fit of the simulated model to the immune response data from [11]. The right panel shows the fit to muscle tissue damage data to soleus muscle taken from [9] and the TA [14].

Even though the model is simple and includes only the key mechanisms of the immune system, it captures the main features of the known dynamics of* mdx* dystrophic muscle pathophysiology, and it manages to reproduce the seemingly unrelated experimental datasets that are reported in the literature as shown in Figure 1. The trajectories of the immune cells in the left panel of Figure 1 fit well the known immune response time course data about macrophages in soleus from [11] and the CD4+ and CD8+ T-cells from [9], while at the same time, the figures in the right panel of Figure 1 show that the model trajectories of the different muscle tissue types are in good agreement with the SOL time course from [15] and the TA time course from [14].

The EDL time course data [15] was not used in the model calibration. However, the model trajectories follow the same patterns as the EDL data, albeit the EDL dynamics are slower as Figure 3 shows.

As mentioned above, the initial counts of the immune cells in the model are set to be equal to those of the wild-type mice. This assumption, however, might not actually be the case for the* mdx* mouse, as the immune response might have started during gestation (prior to birth). To see the effects of onset of the immune response during gestation on the model curves and to explore the sensitivity of the model to these parameters, we varied the values of the three counts and calculated the new trajectories of the 5 variables of the model.  Figure 4 shows the trajectories of macrophages and damaged fibers of 125 different sets of initial values of the three immune cell types. These random values were picked from ranges determined from the available data from wild-type mice. It is clear that these trajectories are qualitatively the same as the corresponding trajectories in Figure 1. This outcome shows that the model is insensitive to the initial counts of immune cells.

### 3.3. Model Prediction

**Figure 2:**
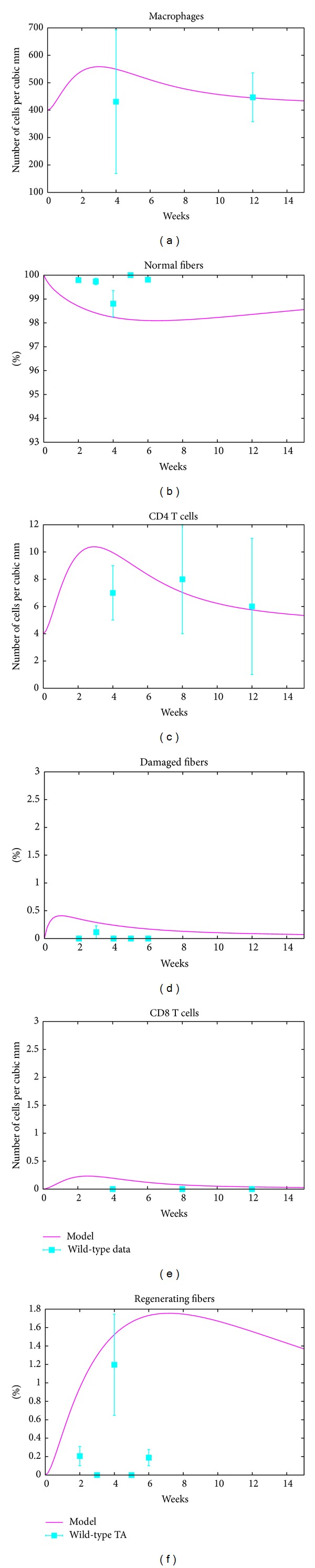
Wild-type mice. The left panel shows the fit of the simulated model to the immune response data from [11]. The right panel shows the fit to muscle tissue data from TA [14].

**Figure 3:**
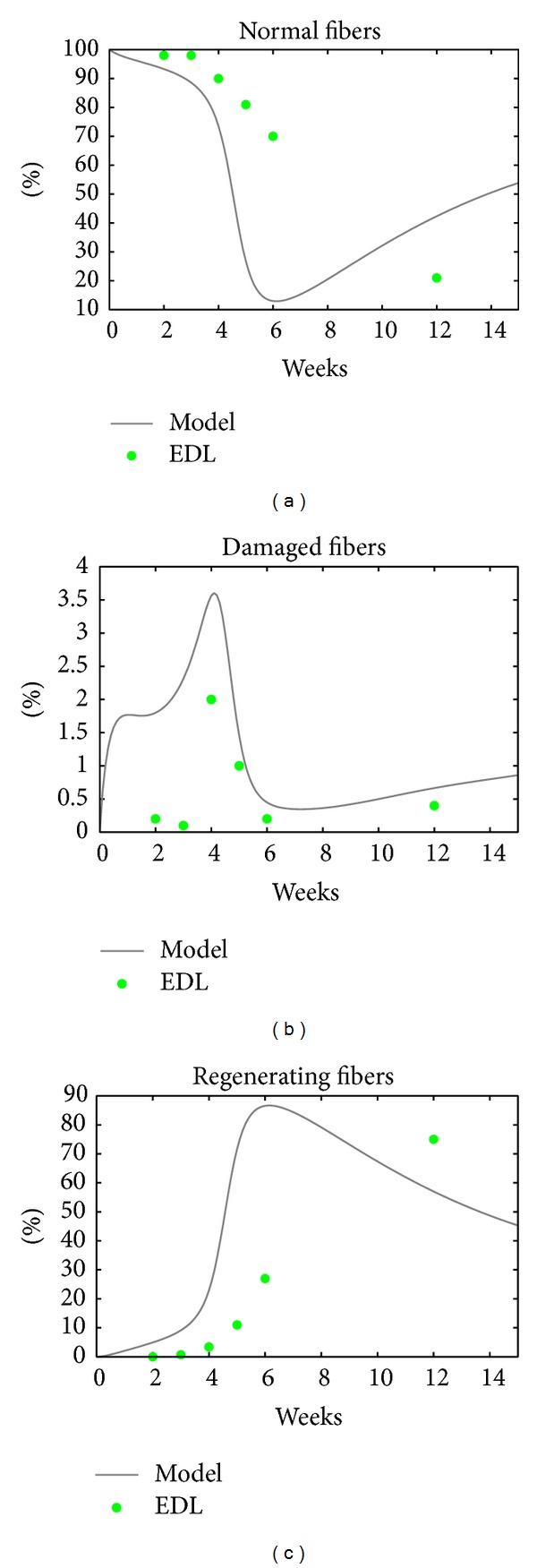
EDL data and the model curves. The model trajectories and the data follow the same pattern, even though the disease time course seems to be slower in EDL than in our model, and this could result because EDL muscle has different physical characteristics from the SOL at TA muscles that were used to fit the model.

**Figure 4:**
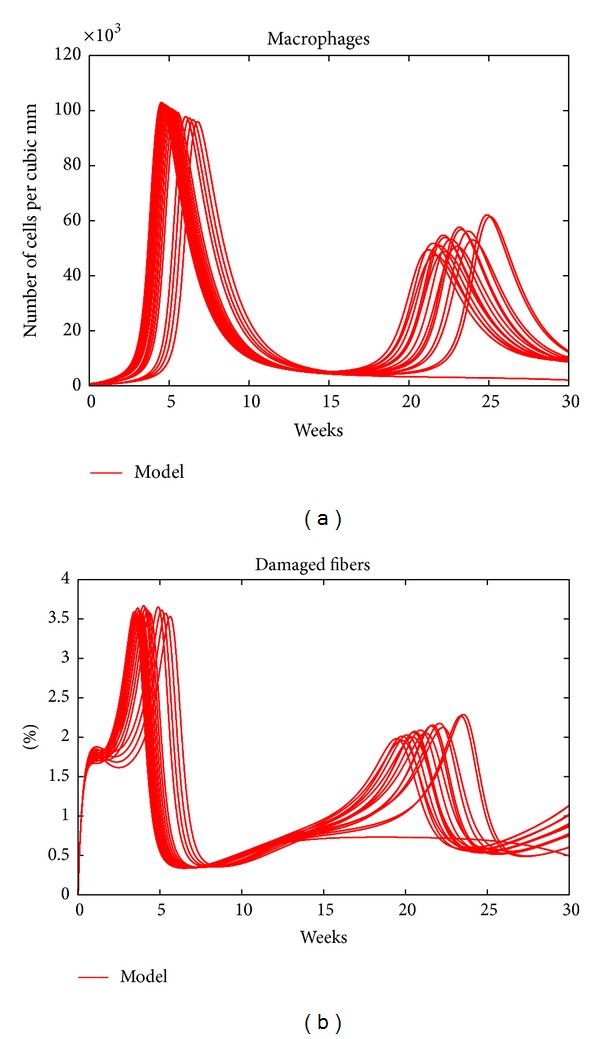
The trajectories of macrophages and damaged fibers starting from 125 different initializations of the immune cell numbers.

As shown in Figure 1, our model trajectories are in good agreement with the available* mdx* mice datasets. Furthermore, the wild-type datasets are in good agreement with our model as shown in Figure 2, where the amplitude *h* of the mechanical damage in the disease-free mice (wild-type) is one tenth of its counterpart in the* mdx* mice.

**Figure 5:**
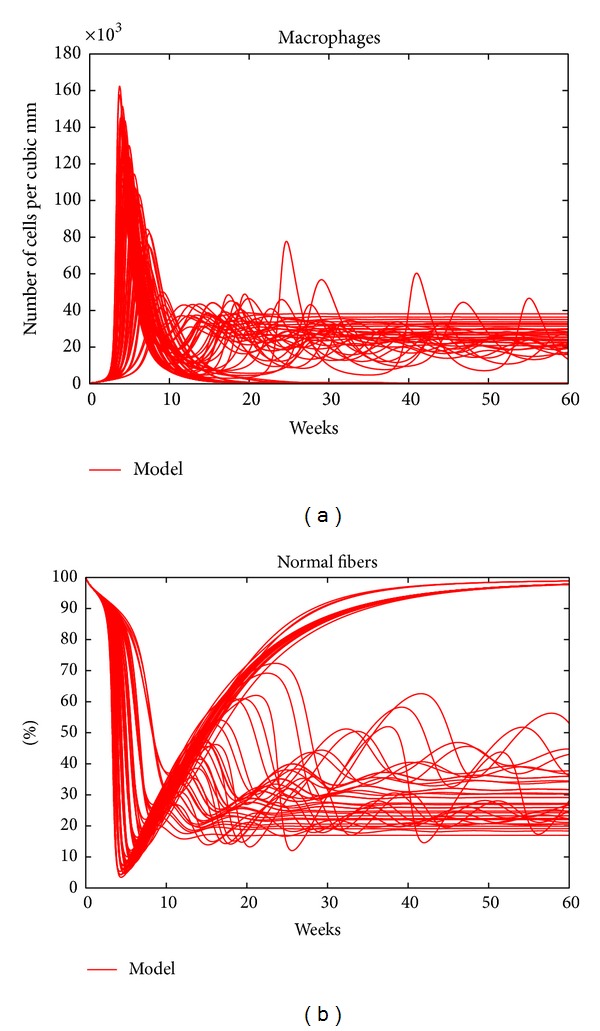
The dynamics of macrophages and normal fibers for a sample of parameter sets from within the specified ranges in Table 2.

To test the model sensitivity to its parameters, we varied the parameters' values from within their ranges as reported in Table 2. For each of the listed parameters, two values were chosen from within the range of that parameter. This approach resulted in more than 1000 parameter sets.  Figure 5 shows the dynamics of macrophages and normal fibers resulting from a sample of these sets. It is clear in Figure 5 that, for the first 7 weeks, the trajectories are qualitatively the same for all parameter sets. However, for the weeks after, and for most of the parameter sets, we see oscillation both in the immune response as well as the different types of muscle fibers.

The literature suggests that “cycles” of muscle degeneration and regeneration contribute to progressive muscle wasting [18–20] which is likely antagonized further by the immune response [8–12]. This relationship suggests a predator-prey-like interaction between the immune system and the tissue, which has been reported in many other diseases [21]. After the dramatic increase of the immune cells and the initial peak of damage in the first 4–8 weeks, the immune cells decrease; muscle degeneration and regeneration cycles are suppressed but continue at low levels. This scenario or mode is captured in Figure 5, by many parameter sets, where oscillation with low amplitude is persistent in the immune response as well as the normal fibers.

For almost all other parameter sets, another mode emerged: the system escapes the oscillatory behavior above and slowly approaches a disease-free state where the percentages of damaged and regenerating fibers will both be very small, and almost all tissue fibers are normal.

This could be the result of a significant decrease of the immune response. In this case, even though regeneration is slow, pathophysiology decreases and degeneration diminishes, which may further suppress the immune response.

The time course for the dystrophic process in* mdx* mice for the first year of life includes muscle degeneration/regeneration cycles between the age of 3 and 10 weeks, followed by a decrease in these cycles and relative muscle stability to the age of ~1 year [19]. The outcomes of the model represented by both modes appear to account for the attenuation of the dystrophic process in mice after the age of ~10 weeks.

Furthermore, the model predicts that the dynamics could switch between the two modes in a response to a change in some of the key parameters, such as the rate at which regenerating fibers become normal.

### 3.4. Immune Cell Depletions

Elevated concentrations of macrophages (>80,000 cells/mm^3^, normal ~1000 cells/mm^3^) have been observed in 4–8 weeks* mdx* mouse muscle but rapidly decrease by 12 weeks [9, 11, 15]. Macrophages have a variety of immunoregulatory and inflammatory functions. They are rich sources of cytokines and nitric oxide, a potent-free radical that can lyse muscle cells. Macrophages are antigen-presenting cells, which may regulate the immune response in dystrophic muscle and could possibly activate T-cells. Antibody-mediated depletion of macrophages, beginning at 6 days old and continuing to 4 weeks in* mdx* mice, resulted in a >75% reduction of injured muscle fibers, suggesting an important role of these cells in the development of lesions [11].

Elevated concentrations of activated CD8+ and CD4+ T-cells are observed in affected muscles of* mdx* mice at the age of 4–8 weeks, but rapidly decrease by 14 weeks (>1200 cells/mm^3^ in* mdx* muscle, normal ~100 cells/mm^3^) [9, 15]. Antibody-mediated depletion of CD8+ or CD4+ T-cells in* mdx* mice, beginning at 6 days old and continuing to 4 weeks of age, resulted in a 75% and 61% reduction in muscle pathology, respectively, for mice at the peak of disease progression [22].

**Figure 6:**
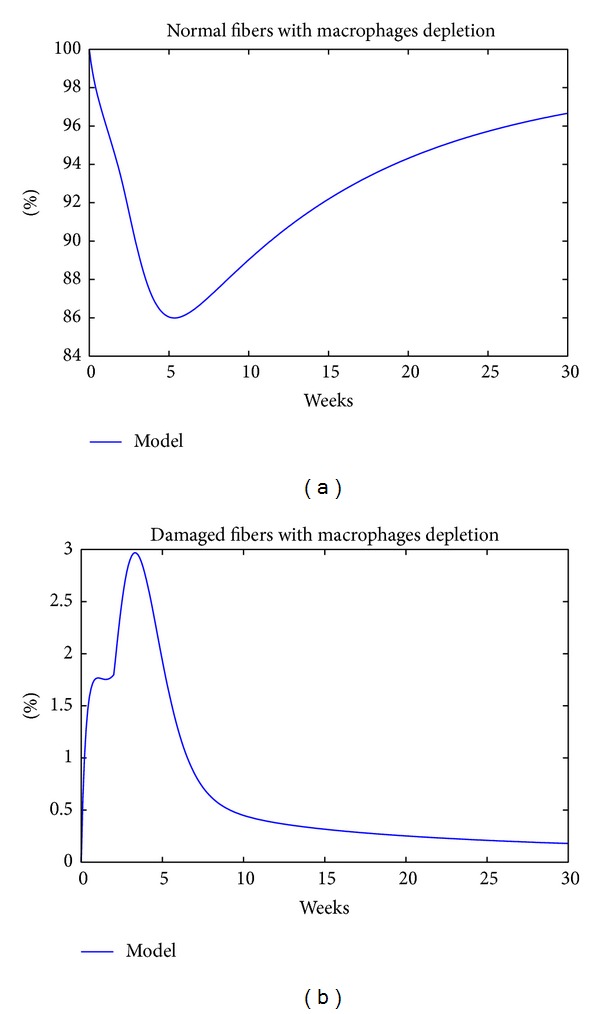
Effect of macrophage depletion on normal and damaged fibers, respectively, in (a) and (b).

According to the model construction, CD8+ T-cells contribute to damage and are produced mainly in response to increased numbers of macrophages; hence, depleting macrophages should reduce the percentage of CD8+ T-cells in the tissue which will do two things: (i) there will be no damage due to CD8+ T-cells, but it also means that there will be no macrophages to clear the damaged cells; (ii) in the model, the macrophage contribution to clearing out the damage is high and so depleting the macrophages will initially increase the damage but then the damage decreases as shown in Figure 6.

Figure 6(a) shows that macrophage depletion in the model resulted in improved muscle tissue with 86% of the muscle fibers characterized as normal. It should be noted here that the percentage of damaged fibers in Figure 6(b) in the tissue is not much lower than the one in Figure 1(d). This outcome may not be surprising since depleting macrophages yields fewer CD8+ T-cells and hence fewer degenerating fibers. At the same time, however, there will be fewer CD4+ T-cells and hence less stimulus to promote the regenerating process.

## 4. Discussion and Conclusions

Immune response is known to play a key role in exacerbating the disease in DMD patients and* mdx* mice. Indeed, a primary treatment for DMD patient is glucocorticoids (GC) whose main effect is the inhibition of numerous inflammatory genes. Even though* mdx* mice show a mild phenotype and do not accurately reflect the severe nature of the human disease, they are considered a reasonable model. In* mdx* mice, several studies demonstrated that depletion of immune cells results in improvement of dystrophic muscle pathophysiology; however, the role and time course of the immune response in the dystrophic process have not been well described. Even though there are time course datasets from different muscles that account for different features of the disease, to our knowledge, there is not a single time course that accounts for both the immune response and muscle pathophysiology in the same muscle type.

We have presented a mathematical model of the immune response to the skeletal muscle wasting and inflammation in* mdx* mice. Our model is simple in the sense that it contains only the basic mechanisms of the immune response and its known interactions with dystrophic muscle. Nonetheless, our model reproduces available data from different experimental studies. Furthermore, the depletions of the immune cells in the model result in an increased percentage of normal fibers as observed in different experiments on* mdx* mice.

**Figure 7:**
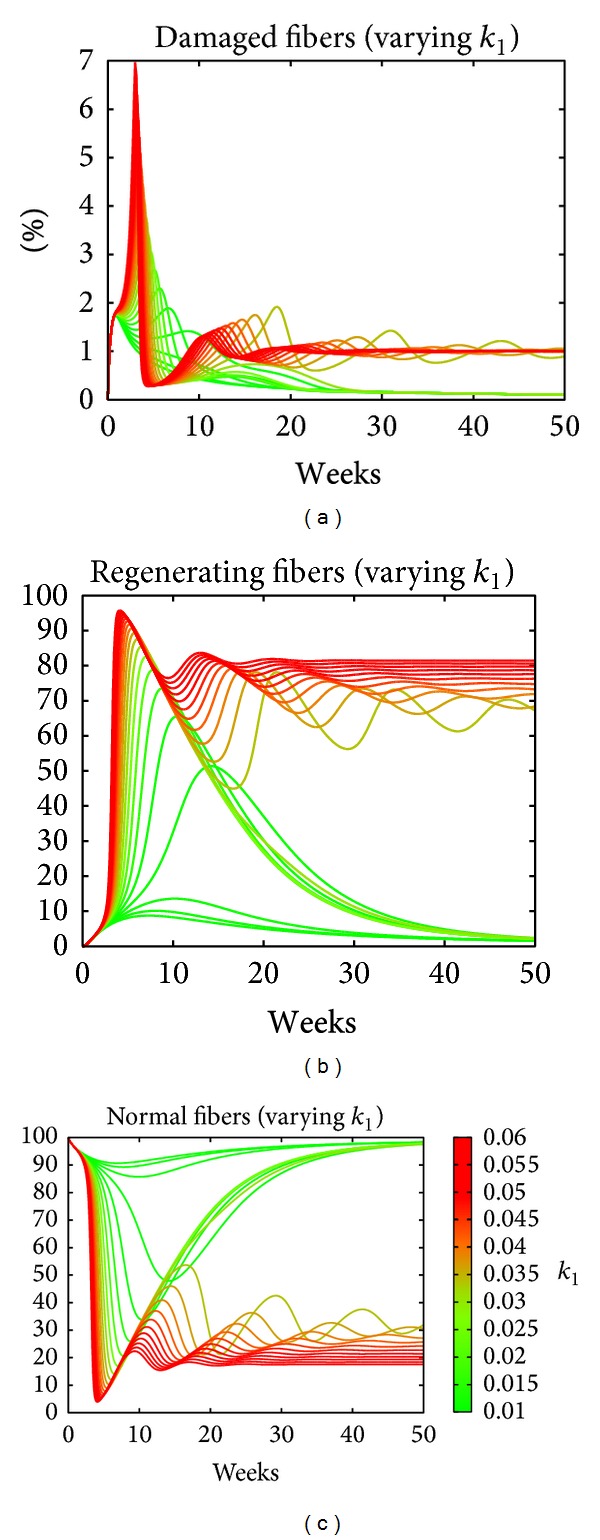
The effect of varying the parameter *k*_1_, while fixing all other parameters, on the trajectory of fibers. As the value of *k*_1_ increases, more tissue goes from damaged (panel (a)) to regenerating (b) and back to normal (c). Interestingly the oscillatory behaviour is found somewhere in between of the two regimes corresponding to *k*_1_ at the lower and at the higher edge of the interval values.

The dynamics of the tuned system shows one main behavior in which the immune system contributes to damage in recurring phases of muscle destruction. However, by changing some crucial parameters' values (in particular parameters *k*_1_, *k*_2_, and *k*_5_) the frequencies of these relapses can be modified eventually obtaining a mode in which the disease is controlled, where damaged fibers regenerate but slowly. The above three parameters are the damaged-driven proliferation rates of CD4+, CD8+ T-cells, and the cytotoxicity of CD4+ T-cells, which are key drivers of the damage in the model.  Figure 7 shows the dependency on *k*_1_ of the trajectories of damaged, regenerating, and normal fibers. As the value of *k*_1_ increases, both the percentage of damaged fibers and the intensity of the immune response increase, leading to an accumulation of regeneration fibers during the period of 5–7 weeks, and this results in an oscillatory behavior where the damage is persistent and around 75 percent of the tissue is regenerating. Similar curves appear when we performed the same analysis using *k*_2_ or *k*_5_.

**Figure 8:**
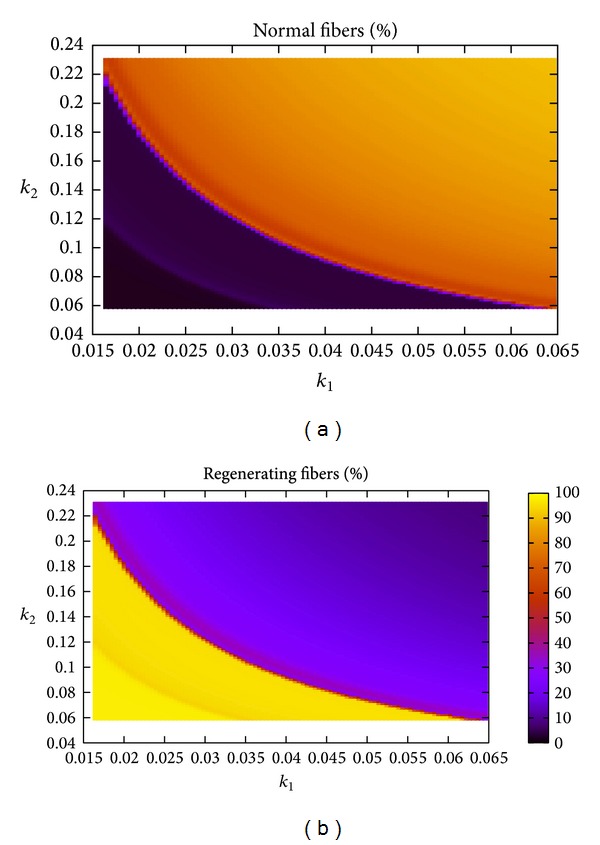
The effect of varying the parameters *k*_1_ and *k*_2_, while fixing all other parameters, on the asymptotic (at week 50) percentages of normal and regenerating fibers. These figures indicate that varying both parameters has a stronger effect than either one of them alone, as indicated by the curve between the dark and bright regions.

The heat maps in Figure 8 show the asymptotic (at week 50) percentages of normal and regenerating when the parameters *k*_1_ and *k*_2_ are changed together. The two main regimes are found for extreme values of the two parameters going from regenerating to normal by increasing both *k*_1_ and *k*_2_. It clearly shows that, by increasing both parameters together, the percentage of normal (regenerating, resp.) would increase (decrease, resp.) at a faster rate than increasing one parameter only. Furthermore, it is easy to see that a small decrease in any of the two parameters would lead to the oscillatory behavior where the majority of the fibers are regenerating.

**Figure 9:**
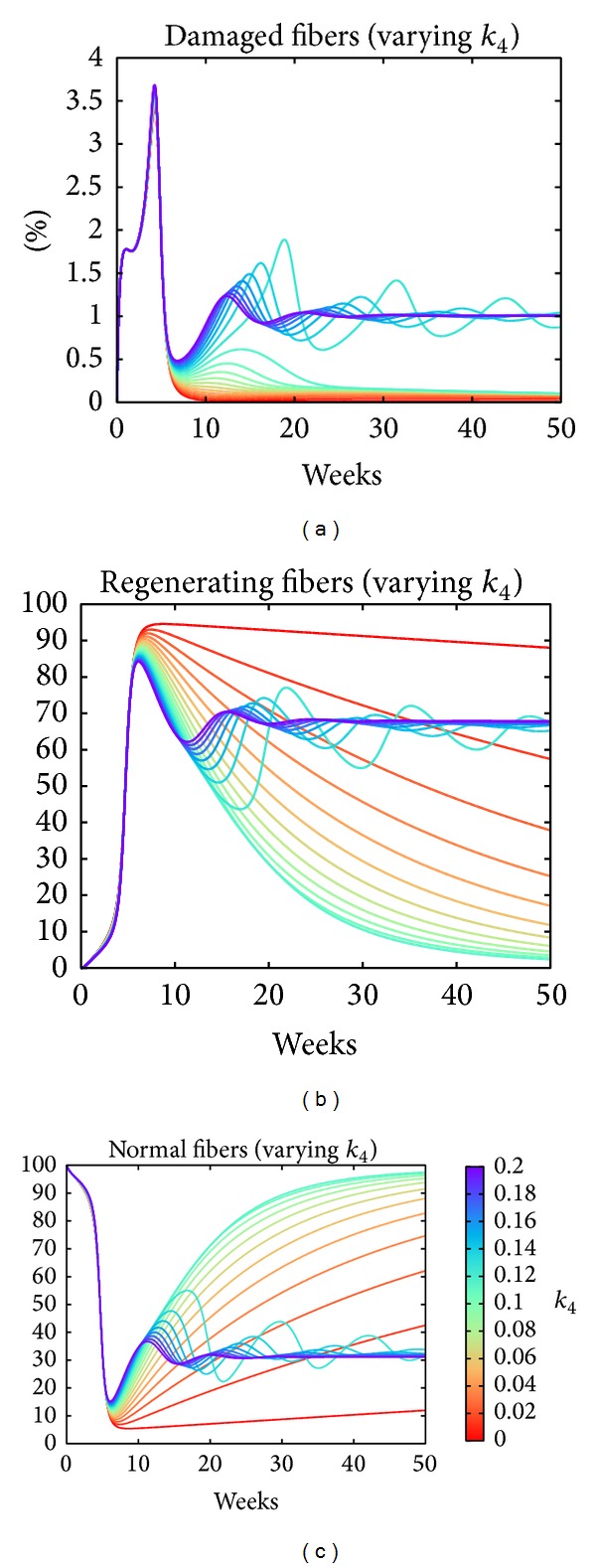
The effect of varying the parameter *k*_4_, while fixing all other parameters, on the trajectories of fibers. As the value of *k*_4_ increases, the percentage of damaged fibers decreases (panel (a)) while the percentage of normal (c) fiber increases and eventually reaches 100% (disease-free state), when *k*_4_ is optimal. Beyond that, however, an oscillatory behaviour appears, where the damage is persistent and most of the tissue is regenerating (b).

Another crucial parameter is the rate at which a regenerating fiber returns, in our model, to morphologically normal muscle fiber,  *k*_4_, which is low indicating a slow regeneration process. Once a fiber is damaged, it could take weeks before it returns to a morphologically normal muscle fiber. This could explain the high percentage of regenerating fibers observed in dystrophic muscles. The model is sensitive to this parameter.  Figure 9 shows how the percentages of damage, regenerating, and normal tissue change when varying *k*_4_. As we increase the value of *k*_4_, the normal tissue asymptotically increases (see Figure 9(c)). However, beyond an optimal value, *k*_4_ ~ 0.1 for which the normal cells are about 100% of the total, the percentage of normal tissues falls to and oscillates around 30%. The other two panels of the same figure show the analogous outcome on damage and regenerating tissue.

In conclusion, although simple, and also considering the limited experimental data, the present model suggests theoretical points of intervention, although the way to achieve the predicted effects through therapeutics may be difficult. This difficulty arises in part because our model does not include additional details such as the relevant signaling pathways, for example, NF-kB, the changes in the population of M1 and M2 macrophages, nor does it keep track of the many potential cytokines and chemokines that are involved.

Nevertheless, the discussion above suggests potential treatment approaches. For example, a treatment approach would decrease antigen presentation by macrophages, thus reducing T-helper activation and consequent cytotoxic stimulation (parameters *k*_1_ and *k*_2_); a second approach attenuates the destruction of normal tissue by cytotoxic T-cells (parameter *k*_5_), while a third approach would drive the generation rate of healthy fibers *k*_4_ to near optimal. In practical terms, however, the suggested therapeutic approaches may not be effective because the model lacks many additional and critical details as noted above. It would be interesting to investigate whether a modification of the model to account for the aforementioned deficits would still identify limiting antigen presentation or cytotoxicity as the main potential candidates for mitigating the immune system effects on the dystrophic pathophysiology.

## Figures and Tables

**Table 1:** The model parameters and their estimated numerical values (in 1 mm^3^ of muscle tissue). Here “c” is for cell and “w” is for week.

Parameter	Description	Value	Unit
*b*_*H*_	Turnover rate of CD4+ T cells	*d*_*H*_ *H*_0_	c w^−1^
*k*_1_	Damage-driven proliferation rate of CD4+ T cells	0.0324139	(cent)^−1^ w^−1^
*d*_*H*_	Death rate of CD4+ T cells	0.83355	w^−1^
*b*_*C*_	Turnover rate of CD8+ T cells	*d*_*C*_ *C*_0_	c w^−1^
*k*_2_	Damage-driven proliferation rate of CD8+ T cells	0.115375	(cent)^−1^ w^−1^
*d*_*C*_	Death rate of CD8+ T cells	1.61511	w^−1^
*b*_*M*_	Turnover rate of macrophages	*d*_*M*_ *M*_0_	c w^−1^
*k*_3_	Infiltration rate of macrophages	0.766576	(cent)^−1^ w^−1^
*d*_*M*_	Death rate of macrophages	0.781155	w^−1^
*k*_4_	Generation rate of healthy fibers	0.123848	w^−1^
*k*_5_	Cytotoxicity degradation rate	4.09948 × 10^−3^	c^−1^ w^−1^
*k*_6_	Cleaning rate by macrophages	3.23097 × 10^−4^	c^−1^ w^−1^
*d*_*D*_	Physiological cleaning rate	1.34671	w^−1^
*σ*	Standard deviation of the initial damage	2.92815	
*m*	The time of the peak of the initial damage	4.22686	
*h*	Proportional to the magnitude of the damage	0.511657	
*H*_0_	The initial number of CD4 T cells	0	c
*C*_0_	The initial number of CD8 T cells	4	c
*M*_0_	The initial number of macrophages	400	c

**Table 2:** Some of the model parameters (column 1), their estimated values (column 2), and, for each parameter, a lower (column 3) and an upper (column 4) bound. To study the sensitivity of the model to a given parameter, we varied the value of that parameter within its range above, and qualitatively examined the resulting dynamics.

Parameter	Value	Min	Max
*k*_1_	0.0324139	0.0160773	0.0643092
*d*_*H*_	0.83355	0.416775	1.6671
*k*_2_	0.115375	0.0577452	0.230981
*d*_*C*_	1.61511	0.807555	3.23022
*k*_3_	0.766576	0.381371	1.52549
*d*_*M*_	0.781155	0.390577	1.56231
*k*_4_	0.123848	0.061924	0.247696
*k*_5_	4.09948 × 10^−3^	2.04974 × 10^−3^	8.19896 × 10^−3^
*k*_6_	3.23097 × 10^−4^	1.61548 × 10^−4^	6.46194 × 10^−4^
*d*_*D*_	1.34671	0.673355	2.69342
